# Comparison of 9-month angiographic outcomes of Resolute zotarolimus-eluting and everolimus-eluting stents in a real world setting of coronary intervention in Korea

**DOI:** 10.1186/1471-2261-13-65

**Published:** 2013-09-08

**Authors:** Joo Myung Lee, Tae-Jin Youn, Jin Joo Park, Il-Young Oh, Chang-Hwan Yoon, Jung-Won Suh, Young-Seok Cho, Goo-Yeong Cho, In-Ho Chae, Dong-Ju Choi

**Affiliations:** 1 Division of Cardiovascular, Department of Internal Medicine, College of Medicine, Seoul National University and Cardiovascular Center, Seoul National University Hospital, Seoul, Korea; 2 Division of Cardiology, Department of Internal Medicine, College of Medicine, Seoul National University and Cardiovascular Center, Seoul National University Bundang Hospital, Seongnam-si, Gyeonggi-do, Korea

**Keywords:** Percutaneous coronary intervention, Drug eluting stent, Late loss, Quantitative coronary angiography, Resolute zotarolimus-eluting stent, Everolimus-eluting stent

## Abstract

**Background:**

Clinical outcomes of new-generation drug-eluting stents (DES), Everolimus-eluting stent (EES) or Resolute zotarolimus-eluting stent (R-ZES), have been reported. However, angiographic follow-up data of new-generation DES are limited, especially in Asians. We investigated the angiographic and clinical outcomes of EES and R-ZES in a real-world setting of Korean patients.

**Methods:**

Angiographic and clinical outcomes of 679 patients (866 lesions) who had been treated with EES or R-ZES from Jun 2008 to May 2010 were evaluated. The primary analysis was to compare in-segment late loss at 9 months and the secondary analyses were to compare the clinical outcomes.

**Results:**

In-segment late loss at 9-month follow-up angiography was 0.23 ± 0.52 mm for EES and 0.29 ± 0.64 mm for R-ZES (p = 0.248). In addition, the rate of binary restenosis did not show between-group differences (5.8% *vs.* 6.8% for EES and R-ZES, respectively, p = 0.716). During a median follow-up of 33 months, there were no significant differences in Kaplan-Meier estimates of target lesion failure (TLF) (7.5% vs. 7.9% for EES and R-ZES, respectively, p = 0.578) and patient-oriented composite outcomes (POCO including all-cause death, any myocardial infarction, and any revascularization, 22.8% vs. 20.1%, p = 0.888). The adjusted hazard ratios for TLF and POCO were 0.875 (95% CI 0.427 - 1.793; p = 0.715) and 1.029 (95% CI 0.642 - 1.650; p = 0.904), respectively, for EES over R-ZES in the propensity score matched group analysis.

**Conclusions:**

In Korean patients undergoing new-generation DES implantation for coronary artery disease, EES and R-ZES showed similar angiographic outcomes at 9 months and comparable clinical outcomes during 2.8 years of median follow-up.

## Background

In the era of drug eluting stents (DES), angiographic and clinical measures of restenosis have been substantially reduced [1-3]. However, long-term clinical outcome analyses have raised concerns about the serious safety problem of stent thrombosis [3-5], which has been known to be associated with allergic and inflammatory reactions to polymers and incomplete strut endothelialization [6-8]. As a consequence, DES with a new-generation polymer were developed, including the Xience V™ everolimus-eluting stent (EES) (Abbott CardioVascular, CA, USA) and the Resolute zotarolimus-eluting stent (R-ZES) (Medtronic CardioVascular, CA, USA). These stents use a cobalt chromium based strut with permanent but biocompatible polymers (poly-vinyl-idene fluoride-co-hexafluoropropylene [PVDF-HFP] and Biolinx, respectively).[9] Recently, results of large scale randomized controlled trials (RCTs) with an ‘all-comers’ design have been reported comparing the efficacy and safety of EES and R-ZES [10-12]. However, angiographic outcomes of these two stents have been limited. In addition, even the RCTs with an ‘all-comers’ design do not include all consecutive patients who are encountered in every day clinical practice [13]. Therefore, the purpose of this study was to evaluate and compare the angiographic and clinical outcomes of EES and R-ZES in the unselected Korean patient population.

## Methods

### Patients population

From June 2008 to May 2010, we prospectively identified 734 patients who were treated by percutaneous coronary intervention (PCI) with either EES or R-ZES for chronic stable coronary artery disease or acute coronary syndrome, including myocardial infarction (MI) with or without ST-segment elevation. Patients who were treated with at least one DES were enrolled in the ‘DES registry of the Seoul National University Bundang Hospital (DES-SNUBH)’ at the catheterization laboratory. There were no restrictions or exclusion criteria for enrollment regarding the reference vessel diameter, total numbers of treated lesion or vessels, numbers of stents implanted, lesion length, referral diagnosis, or comorbidities. Out of 734 patients, 55 patients who were treated with both EES and R-ZES were excluded from the clinical and angiographic outcomes analyses. Therefore, the main analyzed cohort was the remaining 679 patients with 866 lesions (EES 405 patients with 500 lesions; R-ZES 274 patients with 366 lesions) from the registry. Among the main analyzed cohort, 138 patients of EES group (34.2%) and 114 patients of R-ZES group (41.6%) were also enrolled to other Korean multicenter registries (the EXCELLENT and RESOLUTE-Korea registries) under same inclusion and no exclusion criteria [14]. All patients provided written informed consent, and the study protocol was approved by the institutional review board of Seoul National University Bundang Hospital.

### Interventional procedures

Coronary interventions were performed according to current standard techniques. The choice of the stent, predilatation, post-stenting adjunctive balloon inflation, and the use of intravascular ultrasound or glycoprotein IIb/IIIa inhibitors were all left to the operators’ discretion. All patients received at least 100 mg of aspirin before the procedure. A loading dose of 300 to 600 mg of clopidogrel was administered to all patients who were not on clopidogrel prior to the procedure. After the procedure, all patients were given aspirin (at least 100 mg/day) indefinitely and clopidogrel (75 mg/day) for at least 1 year after index procedure. During the procedure, unfractionated heparin at a dose of 70 to 100U per kilogram of body weight was administered to achieve and maintain an activated clotting time of more than 250 seconds.

### Clinical and angiographic follow-up and quantitative coronary angiography

Clinical follow-up after PCI was recommended at 1 month, 6 months, and 1 year and annually thereafter. Angiographic follow-up was routinely recommended at 9 months, post-PCI or earlier if patients complained of any ischemic symptoms or if noninvasive evaluation suggested the presence of ischemia. The follow-up angiography was performed systematically and in a routine manner, unless the patient rejected the follow-up procedure or the patient’s condition was not suitable to undergo coronary angiography. All patients enrolled in this registry provided a written informed consent not only for the enrollment but also for the follow-up angiography. Coronary angiography obtained at baseline, immediately postprocedure, and at follow-up were analyzed quantitatively (by quantitative coronary angiography [QCA]) using the Cardiovascular Angiography Analysis System (CAAS) II (Pie Medical Imaging, Maastricht, Netherlands) by an experienced technician who was not aware of the study purpose. The minimum lumen diameter (MLD), reference vessel diameter, percent stenosis, acute gain (defined as the difference in MLD before and after stent implantation), late lumen loss (defined as the difference between the postprocedure and follow-up MLD), and binary restenosis (defined as stenosis of 50% or more at follow-up angiography) were measured [15]. All QCA measurements of the target lesion were obtained in the in-stent zone, and over the entire segment including the stent and its 5-mm proximal and distal margins (in-segment zone).

### Definitions and outcomes analysis

The stent-related clinical outcome was target lesion failure (TLF), defined as a composite of cardiac death, MI (not clearly attributed to a nontarget vessel), or a clinically indicated target lesion revascularization by percutaneous or surgical methods [16]. The patient-related clinical outcome was patient-oriented composite outcome (POCO) which included all-cause mortality, any MI (including nontarget vessel territory), and any revascularization (including all target and nontarget vessels, regardless of percutaneous or surgical methods) [16]. Other clinical outcomes, including MI, stent thrombosis, target lesion revascularization, and target vessel revascularization were defined as according to Academic Research Consortium (ARC) definitions [16]. Patients with complex lesions (considered an off-label indication for use of both DES) were defined as having at least one of the following characteristics: serum creatinine concentration ≥140 umol/L (1.6 mg/dL); left ventricular ejection fraction (LVEF) < 30%; an acute MI within the previous 72 hours; more than one lesion per vessel; two or more vessels treated with a stent; a lesion greater than 27 mm; or a bifurcated lesion, bypass graft, in-stent restenosis, unprotected left main coronary artery, presence of thrombus, or total occlusion [11,17].

### Statistical analysis

Statistical analyses were performed in three parts. The primary analysis was to compare the angiographic outcomes at 9 months and the secondary analyses were to compare the clinical outcomes. Comparison of angiographic outcomes between two groups was performed in the crude population. Clinical outcome analysis was perfomed on 1) the crude population and 2) the propensity score matched population. Event rates of clinical outcomes were expressed as incidence density (i.e. Kaplan-Meier estimates) and the log-rank or Breslow test was used to compare between-group differences. For the subgroup analysis of TLF, Cox proportional hazard model was used to calculate the hazard ratio of EES compared with R-ZES, and interaction p values between treatment and each subgroup. Since differences in baseline characteristics could impact the development of clinical outcomes, a 1:1 matched analysis without replacement was performed using propensity score which was calculated from the model with 23 covariates (Additional file 1: Table S1). The log-odds of the probability that a patient received an R-ZES were modeled as a function of the confounders. A caliper width of 0.6 SDs was used because this value has been shown to eliminate almost 90% of the bias in the observed confounders [18]. Success of the propensity score matching was assessed by calculating percentage standardized differences of the baseline characteristics. A less than 10% difference supports the assumption of a balance between matched groups [19]. Propensity score adjusted stratified Cox proportional hazard regression model was fitted to compare the clinical outcomes of the matched EES and R-ZES groups. All probability values were two-sided and p-values < 0.05 were considered statistically significant. The statistical package SPSS, version 18.0 (SPSS Inc., Chicago, IL, USA) and R programming language, version 2.12.2 (R Foundation for Statistical Computing) were used for statistical analyses.

## Results

### Baseline clinical and angiographic characteristics

**Figure 1:**
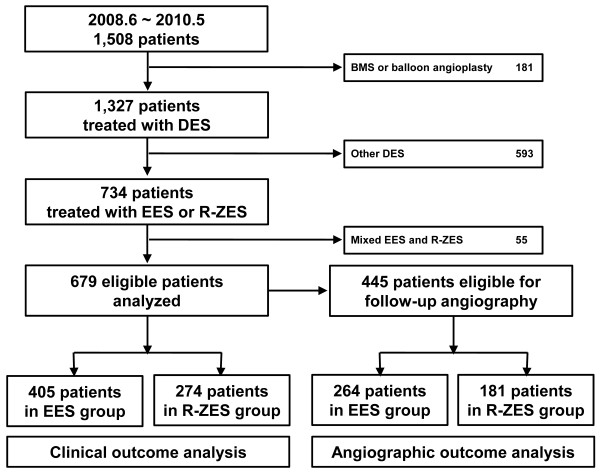
**Flow of participants diagram.** Abbreviations: *EES*, everolimus-eluting stent; *R-ZES*, Resolute zotarolimus eluting-stent.

During the study period, a total of 1,508 patients received PCI in our institution. One hundred and eighty-one patients were treated with bare-metal stent or balloon angioplasty. Of the remaining 1,327 patients who were treated with DES, 734 patients (55.3%) were treated with EES or R-ZES. Fifty-five patients were excluded since they were treated with EES and R-ZES simultaneously. The flow of participants for the study is presented in Figure 1. The cohort used for the main analysis consisted of 679 patients with 866 lesions (EES 500 lesions/405 patients, R-ZES 366 lesions/274 patients). The baseline clinical characteristics were similar in both groups, except that the proportion of patients with multivessel disease was higher in R-ZES group (Table 1). A total of 501 of 679 (73.8%) patients were classified as complex, which was distributed similarly in both group. EES was more frequently implanted into the left main coronary lesion and the in-stent restenosis lesion than R-ZES; however, R-ZES was more often used for small vessel lesion. As a consequence, the mean stent diameter was significantly smaller in the R-ZES group. The mean in-stent and in-segment MLDs after the procedure were also smaller in R-ZES group than in EES group, despite the similar acute gains in the two groups (Table 2).

**Table 1:** Baseline clinical characteristics of crude population^*^

	**EES (N = 405)**	**R-ZES (N = 274)**	**P value**
Age, years	63.9 ± 11.8	64.9 ± 11.9	0.264
Male	297 (73.3%)	197 (71.9%)	0.680
**Coexisting condition**			
Diabetes mellitus	157 (38.8%)	101 (36.9%)	0.616
Hypertension	283 (69.9%)	172 (62.8%)	0.056
Dyslipidemia	140 (34.6%)	91 (33.2%)	0.714
Cerebrovascular disease	35 (8.7%)	18 (6.6%)	0.325
Peripheral artery disease	9 (2.2%)	9 (3.3%)	0.398
Chronic renal failure	15 (3.7%)	14 (5.1%)	0.374
**Cardiovascular risk factors**			
Current smoker	103 (25.4%)	77 (28.1%)	0.439
Previous PCI	69 (17.0%)	40 (14.6%)	0.396
Previous CABG	5 (1.2%)	5 (1.8%)	0.531
Previous MI	33 (8.1%)	25 (9.1%)	0.842
**Clinical indications**			
Stable angina	189 (46.7%)	124 (45.3%)	0.717
Unstable angina	99 (24.4%)	64 (23.4%)	0.745
Acute myocardial infarction	117 (28.9%)	86 (31.4%)	0.485
Emergency PCI for acute STEMI	41 (10.1%)	34 (12.4%)	0.351
Left ventricular ejection fraction	57.11 ± 10.40	56.25 ± 11.71	0.374
Severe LV dysfunction (LVEF < 30%)	6 (1.5%)	7 (2.6%)	0.317
Multivessel disease	275 (67.9%)	207 (75.5%)	0.031
Complex patient^†^	293 (72.3%)	208 (75.9%)	0.328

^*^ Data are number (%), unless otherwise indicated. Plus-minus values are means ± SD.

^†^ Complex patient : Complex patients (= off label use) were defined as having at least one of the following characteristics: serum creatinine concentration of 140 umol/L (1.6 mg/dL) or more; left ventricular ejection fraction < 30%; an acute myocardial infarction within the previous 72 hours; more than one lesion per vessel; two or more vessels treated with a stent; a lesion longer than 27mm; or bifurcated lesion, bypass graft, in-stent restenosis, unprotected left main coronary artery, presence of thrombus, or total occlusion.

*Abbreviations*: *CABG* coronary artery bypass graft, *EES* everolimus-eluting stent, *F/U* follow-up, *IQR* interquartile range, *LV* left ventricle, *LVEF* left ventricular ejection fraction, *MI* myocardial infarction, *PCI* percutaneous coronary intervention, *R-ZES* Resolute zotarolimus eluting-stent, *STEMI* myocardial infarction with ST-segment elevation.

**Table 2:** Baseline lesional characteristics and quantitative coronary angiography data of crude population^*^

	**EES (N = 500)**	**R-ZES (N = 366)**	**P value**
**Before index procedure**			
Target vessel location			0.509
Left main	32 (6.4%)	11 (3.0%)	
LAD	252 (50.4%)	168 (45.9%)	
LCX	109 (21.8%)	95 (26.0%)	
RCA	135 (27.0%)	100 (27.3%)	
Type B2 or C lesions^†^	315 (63.0%)	227 (62.0%)	0.769
In-stent restenosis	39 (7.8%)	15 (4.1%)	0.032
Chronic total occlusion	14 (2.8%)	20 (5.5%)	0.052
Bifurcation^‡^	31 (6.2%)	31 (8.5%)	0.201
Small vessel^§^	169 (33.8%)	167 (45.6%)	0.001
Long lesion^¶^	102 (20.4%)	69 (18.9%)	0.572
Lesion length, mm	20.63 ± 9.89	21.10 ± 10.32	0.530
Reference diameter, mm	2.90 ± 0.59	2.79 ± 0.58	0.012
Minimum lumen diameter, mm	0.75 ± 0.49	0.72 ± 0.51	0.352
Percent stenosis, %	79.60 ± 15.35	78.03 ± 17.05	0.162
**After index procedure**			
Number of stents/lesion	1.23 ± 0.47	1.20 ± 0.43	0.226
Total stent length/lesion, mm	27.83 ± 13.33	28.16 ± 12.55	0.717
Pressure deployment, atm	15.02 ± 5.94	15.12 ± 4.74	0.783
Mean stent diameter/lesion, mm	3.11 ± 0.45	2.91 ± 0.40	< 0.001
Minimum lumen diameter, mm			
In-stent	2.61 ± 0.47	2.51 ± 0.43	0.001
In-segment	2.60 ± 0.48	2.49 ± 0.45	0.001
Percent stenosis, %			
In-stent	12.85 ± 7.00	11.71 ± 7.11	0.019
In-segment	12.35 ± 7.32	11.38 ± 7.58	0.063
Acute gain, mm			
In-stent	1.88 ± 0.61	1.80 ± 0.55	0.094
In-segment	1.86 ± 0.63	1.79 ± 0.57	0.128

^*^ Data are number (%), unless otherwise indicated. Plus-minus values are means ± SD.

^†^ Type B2 or C lesions according to ACC/AHA classification.

^‡^ Bifurcation means bifurcated lesion that have been treated solely by drug-eluting stents.

^§^ Small vessel denotes lesion with reference diameter < 2.75 mm.

^¶^ Long lesion denotes lesion with length ≥ 28 mm.

*Abbreviations*: *EES* everolimus-eluting stent, *LAD* left anterior descending artery, *LCX* left circumflex artery, *RCA* right coronary artery, *R-ZES* Resolute zotarolimus eluting-stent.

### Angiographic outcomes and at 9 months

Angiographic follow-up at 9 months was available in 445 patients with 564 lesions (65.5%) (Figure 1). Baseline clinical and angiographic characteristics of the patient cohort undergoing QCA analysis at 9 months are listed in Additional file 1: Table S2 and were similar between groups. In-segment late loss was 0.23 ± 0.52 mm for EES and 0.29 ± 0.64 mm for R-ZES (p = 0.248). In-stent late loss showed similar findings (0.24 ± 0.53 mm for EES vs. 0.29 ± 0.58 mm for R-ZES, p = 0.267). In addition, the rate of binary restenosis (5.8% *vs.* 6.8%, p = 0.716) did not show between-group differences, despite significantly smaller reference vessel diameter in the R-ZES gropup (2.88 ± 0.60 mm vs. 2.77 ± 0.50 mm, p = 0.038) (Table 3).

**Table 3:** Angiographic outcomes with quantitative coronary angiography data at 9 months follow-up^*^

	**EES (Lesion n = 324)**	**R-ZES (Lesion n = 240)**	**P value**
**Number of patients**	**264 (65.2%)**	**181 (66.1%)**	**0.869**
**Before index procedure**			
Lesion length, mm	20.33 ± 10.04	20.89 ± 8.89	0.528
Reference diameter, mm	2.88 ± 0.60	2.77 ± 0.50	0.038
Minimum lumen diameter, mm	0.73 ± 0.50	0.71 ± 0.53	0.769
Percent stenosis, %	80.73 ± 14.79	77.64 ± 17.24	0.024
**After index procedure**			
Total stent length, mm	27.03 ± 12.91	28.61 ± 12.88	0.153
Number of stents/lesion	1.20 ± 0.45	1.20 ± 0.43	0.889
Minimum lumen diameter, mm			
In-stent	2.60 ± 0.48	2.53 ± 0.43	0.061
In-segment	2.59 ± 0.50	2.50 ± 0.46	0.030
Percent stenosis, %			
In-stent	13.03 ± 6.92	11.17 ± 6.26	0.001
In-segment	12.60 ± 7.20	11.27 ± 7.53	0.037
Acute gain, mm			
In-stent	1.89 ± 0.64	1.83 ± 0.56	0.284
In-segment	1.87 ± 0.67	1.80 ± 0.60	0.237
**Follow-up procedure**			
Minimum lumen diameter, mm			
In-stent	2.37 ± 0.66	2.23 ± 0.64	0.017
In-segment	2.36 ± 0.66	2.20 ± 0.66	0.006
Percent stenosis, %			
In-stent	20.65 ± 17.52	20.50 ± 19.23	0.925
In-segment	19.41 ± 17.30	20.68 ± 19.19	0.431
Late lumen loss, mm			
In-stent	0.24 ± 0.53	0.29 ± 0.58	0.267
In-segment	0.23 ± 0.52	0.29 ± 0.64	0.248
Loss index			
In-stent	0.19 ± 0.78	0.09 ± 0.57	0.129
In-segment	0.15 ± 0.81	0.07 ± 0.99	0.093
Binary restenosis, %			
In-stent	18 (5.8%)	15 (6.8%)	0.716
In-segment	17 (5.6%)	15 (6.9%)	0.582

^*^ Among a total of 679 patients, angiographic follow-up was available in 65.5% of patients (445 patients with 561 lesions). Data are number (%), unless otherwise indicated. Plus-minus values are means ± SD.

*Abbreviations:EES* everolimus-eluting stent, *R-ZES* Resolute zotarolimus eluting-stent.

### Clinical outcomes up to 3 years of follow-up

**Figure 2:**
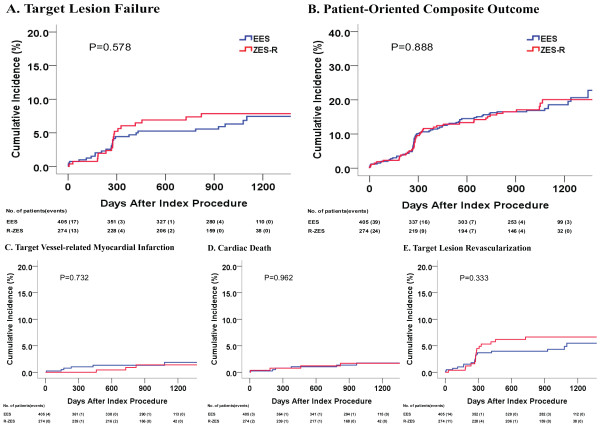
**Survival analysis for target lesion failure and patient-oriented composite outcome up to 3 years of follow-up. A)** Target lesion failure. **B)** Patient-oriented composite outcome. **C)** Target vessel myocardial infarction **D)** Cardiac death **E)** Target lesion revascularization. Target lesion failure defined as a composite of cardiac death, myocardial infarction (not clearly attributed to a nontarget vessel), or clinically indicated target lesion revascularization by percutaneous or surgical methods. Patient-oriented composite outcome included all-cause mortality, any myocardial infarction (includes nontarget vessel territory), and any revascularization (includes all target and nontarget vessel, regardless of percutaneous or surgical methods). Log rank p value or Breslow p value were presented. Abbreviations: *EES*, everolimus-eluting stent; *R-ZES*, Resolute zotarolimus eluting-stent.

The median follow-up duration after index procedure was 1014 days (33 months, interquartile range 27.0–38.0 months). There were no significant differences in Kaplan-Meier estimates of TLF (7.5% in the EES group vs. 7.9% in the R-ZES group, p = 0.578) or POCO (22.8% vs. 20.1%, p = 0.888). A total of 9 patients developed ARC-defined definite or probable stent thrombosis. Kaplan-Meier estimates of definite or probable stent thrombosis were not significantly different between the groups (1.5% *vs.* 1.8%; p = 0.741) (Figure 2 and Table 4). Detailed descriptions of the cases are presented in Additional file 1: Table S3.

**Table 4:** Clinical outcomes in crude population up to 3 years of follow-up (Kaplan-Meier estimates and log-rank p value)^*^

	**EES (N = 405)**	**R-ZES (N = 274)**	**P value**
All cause death	4.6% (16)	3.9% (9)	0.753
Cardiac death	1.7% (6)	1.7% (4)	0.962
Any myocardial infarction	2.1% (7)	1.9% (4)	0.858
Target vessel	1.8% (6)	1.4% (3)	0.732
Non Target vessel	0.3% (1)	0.4% (1)	0.760
MI due to ST	1.0% (3)	1.4% (3)	0.564
Target lesion revascularization	5.6% (18)	6.6% (16)	0.333
Target vessel revascularization	7.7% (26)	8.3% (20)	0.539
Any revascularization	19.2% (54)	17.4% (37)	0.603
Target lesion failure^†^	7.5% (25)	7.9% (19)	0.578
Patient-oriented composite outcome^‡^	22.8% (69)	20.1% (44)	0.888
Definite ST	1.3% (4)	0.9% (2)	0.789
Acute (0–1 day)	0.0%	0.0%	NA
Subacute (2–30 days)	0.5% (2)	0.0%	0.249
Late (31–360 days)	0.3% (1)	0.0%	0.418
Very late (≥ 361 days)	0.5% (1)	0.9% (2)	0.296
Probable ST	0.3% (1)	0.9% (2)	0.344
Acute (0–1 day)	0.3% (1)	0.0%	0.411
Subacute (2–30 days)	0.0%	0.4% (1) ^§^	0.221
Late (31–360 days)	0.0%	0.0%	NA
Very late (≥ 361 days)	0.0%	0.5% (1)	0.215
Definite/Probable ST	1.5% (5)	1.8% (4)	0.741

^*^ Clinical outcomes were presented as cumulative incidence (Kaplan-Meier estimates, %) of primary and secondary clinical outcomes calculated. Numbers of total events are presented in parentheses. P values are log-rank p value or Breslow p value.

^†^ Target lesion failure defined as a composite of cardiac death, myocardial infarction (not clearly attributed to a nontarget vessel), or clinically indicated target lesion revascularization by percutaneous or surgical methods at 3 years.

^‡^ Patient-oriented composite outcomes included all-cause mortality, any myocardial infarction (includes nontarget vessel territory), and any revascularization (includes all target and nontarget vessel, regardless of percutaneous or surgical methods).

^§^ Subacute stent thrombosis, presented with sudden cardiac arrest which occurred at 6 days after percutaneous coronary intervention.

*Abbreviations*: *EES* everolimus-eluting stent, *MI* myocardial infarction, *R-ZES* Resolute zotarolimus eluting-stent, *ST* stent thrombosis, *EES* everolimus-eluting stent.

### Subgroup analysis

**Figure 3:**
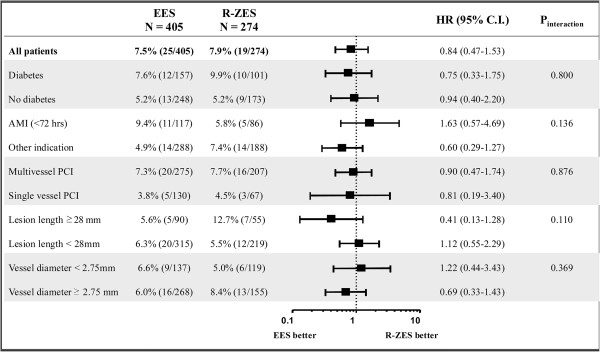
**Subgroup analysis.** Abbreviations: *EES*, everolimus-eluting stent; *R-ZES*, Resolute zotarolimus eluting-stent; *HR*, hazard ratio; *CI*, confidence interval; *AMI*, acute myocardial infarction; *PCI*, percutaneous coronary intervention.

Exploratory subgroup analyses regarding TLF were performed according to the presence of diabetes, acute myocardial infarction (< 72 hours), multivessel PCI, long lesion (≥ 28 mm), and small vessel (< 2.75 mm). There were no significant differences between EES and R-ZES, and the results were consistent across all subgroups, with no significant interaction p values (Figure 3).

### Propensity score matched group analysis

Matching by propensity score with caliper width of 0.6 SDs yielded 249 EES patients matched to 249 R-ZES patients. Standardized differences of baseline clinical and angiographic characteristics were less than 10%, and both groups were more balanced than before matching, with the exception of bifurcation lesion (percent standardized difference 12.91%, Additional file 1: Table S4). The comparable incidences of clinical outcomes were corroborated in propensity score matched group analysis. The adjusted hazard ratio for TLF and POCO were 0.875 (95% CI 0.427 - 1.793; p = 0.715) and 1.029 (95% CI 0.642 - 1.650; p = 0.904), respectively, for EES over R-ZES (Table 5). Among the propensity-score matched population, 325 patients (65.3% of 498 patients) performed 9-month follow-up angiography. Angiographic outcomes including in-stent and in-segment late loss, and the rate of binary restenosis were comparable between the two groups (data not shown).

**Table 5:** Clinical outcomes during follow-up period in the propensity score matched groups (249 pairs)

	**EES**	**R-ZES**	Adjusted HR^*^	**95% CI**	**P value**
All cause death	4.9 (11)	3.7 (8)	1.833	0.678-4.957	0.232
Cardiac death	2.2 (5)	1.9 (4)	1.667	0.398-6.974	0.484
Any myocardial infarction	2.7 (5)	2.0 (4)	1.000	0.202-4.955	>0.999
Target vessel	2.2 (4)	1.6 (3)	1.500	0.251-8.977	0.657
Target lesion revascularization	5.7 (10)	7.2 (16)	0.533	0.226-1.258	0.151
Target vessel revascularization	7.4 (14)	9.1 (20)	0.706	0.337-1.478	0.356
Any revascularization	22.3 (35)	18.6 (36)	0.833	0.490-1.417	0.501
Target lesion failure	8.4 (16)	8.6 (19)	0.875	0.427-1.793	0.715
Patient-oriented outcome	26.4 (46)	21.1 (42)	1.029	0.642-1.650	0.904

^*^ Hazard ratio was calculated with stratified Cox proportional hazard regression, values are hazard ratio of EES compared with R-ZES.

*Abbreviations*: *CI* confidence interval, *EES* everolimus-eluting stent, *HR* hazard ratio, *R-ZES* Resolute zotarolimus-eluting stent.

## Discussion

This observational study compared the clinical and angiographic outcomes of two new-generation DES—the Xience V EES and the Resolute ZES—in an unselected patient population without exclusion criteria. There was no significant difference in the primary angiographic outcome, in-segment late loss at 9 months, between the two stent groups. The stent- and patient-related clinical outcomes (TLF and POCO, respectively) were comparable up to 3 years of follow-up, which were corroborated by the similar results from the propensity score matched cohort.

In our study, angiographic follow-up data were available in a relatively larger proportion of patients (445 among 679 patients, 65.5%) compared with previous RCT analyzing the angiographic outcomes of EES and R-ZES in all-comers population (RESOLUTE All Comers trial (272 patients among 2292 patients, 11.9%) [10]. To the best of our knowledge, this study includes the largest angiographic cohort for the comparison of EES and R-ZES. Although there were several significant differences in baseline clinical and angiographic characteristics between the two groups, which is an inherent limitation of non-randomized studies, the angiographic outcomes, not only the binary restenosis but also the in-stent or in-segment late loss at 9 months, were comparable. Our angiographic follow-up data, performed in a relatively large patient population with complex coronary anatomy, support the previous result of RESOLUTE ALL Comers trial, which demonstrated no difference between EES and R-ZES in inhibitory effect on neointimal hyperplasia [10]. With regard to the differences in baseline clinical and angiographic characteristics between the two groups, selection bias might stem from the operator’s experiences with the stent utilized. We postulate that the preference for EES in left main and restenotic lesions may be explained by longer experience with EES, which has been introduced earlier than R-ZES. In contrast, the previous experience with ZES (Endeavor® Splint) with better deliverability compared to the first generation DES may have contributed to favoring R-ZES in small and multivessel disease.

Previously, RESOLUTE All Comers trials reported that R-ZES and EES showed similar safety and efficacy outcomes up to 2-year follow-up [10,11]. Although well-designed RCTs are the gold standard to evaluate safety and efficacy of therapeutic options, these may not reflect the actual clinical practice due to highly selective inclusion criteria. Even RESOLUTE All Comers trial—a RCT with an ‘all-comers’ design—was not able to enroll all consecutive patients, but included only 44% of them [13]. While we prepared this report, the result of TWENTE trial was published, and they also reported the similar clinical outcomes for both stent types at 1 year [11]. Although, they enrolled more than 80% of all eligible patients and 77% of the enrolled patients were with off-label indication for DES, they excluded acute ST-segment elevation MI patients. In this study, we tried to evaluate and reflect routine ‘real world’ clinical practice as much as possible and the patients were enrolled at the time of the index procedure without exclusion criteria. As a result, a variety of patients with differing clinical and angiographic characteristics, such as acute MI, severe LV dysfunction, left main coronary artery disease, small vessel disease, long lesions, or in-stent restenosis lesions, were included for this analysis. Notably, the overall proportion of acute coronary syndrome was 53.9% (366/679 patients), those with diabetes mellitus was 38.0% (258/679 patients), and the majority of the population (73.8%) had at least one criterion for complex patients or lesional characteristics. The large proportion of high-risk patients and lesions implies that our study population well reflects real-world practice in Korea without any exclusion or restriction.

It is noteworthy that, in a 2-year follow-up of the RESOLUTE All Comers trial, Silber et al. reported substantially higher rates of patient-related outcome than stent-related outcomes [11]. In agreement with the above study, we also noticed substantial difference between the rates of patient-oriented composite outcomes and stent-related outcomes (TLF). These findings emphasize the importance of secondary prevention of cardiac risk factors and overall medical management of non-cardiac comorbidities [11].

### Limitations

Some limitations of this study should be considered. First, this study was a non-randomized observational study using a single center registry data with a relatively small number of patients and was not sufficiently powered to compare clinical outcomes between two stent groups due to limited sample size and low event rates. Second, even though we used propensity score matching and stratified Cox proportional hazard regression modeling to minimize the allocation bias and control for potential confounding variables, the possibilities of uncontrolled and unknown confounding factors need to be considered. Third, because data were from observational registries, the clinical events may not have been captured with scrutiny and patient follow-up may not have been as tight as would be in RCTs. Although we analyzed clinical outcomes up to 3 years of follow-up, a considerable number was lost to follow-up during the second year of follow-up. This can also be another source of bias and may explain lower TLF rate compared with that of RESOLUTE All Comer study at 2 year. However, censoring distributions of the two groups for the clinical outcomes were similar and little influence on the comparisons of two groups is expected. Fourth, the criteria of complex patients or lesional characteristics could not include homogeneous patient population. For example, a patient with severe renal insufficiency which has (at the same time) an acute MI due to left main disease is really different from a patient which receives a stent in an obtuse marginal artery because of in-stent re-stenosis, but both patients would be represented as the same complex group. Fifth, QCA analysis was not performed by an independent core laboratory, but rather by a single analyst. Although the analyst was blinded to the allocated stent group, systematic error cannot be completely excluded. In addition, there were no available data about intravascular ultrasound guidance PCI or the use of post-adjunctive balloon which is important for the optimal procedure. At last, a relatively high number of clinical events occurred around the ninth month during the follow-up, when the follow-up angiography was performed. It has been demonstrated that mandatory angiographic follow-up increases rates of revascularization [20]. We also speculate that the so-called ‘oculo-stenotic reflex’ may have contributed to the revascularization rates in this study. Therefore the present results cannot be directly extrapolated to routine clinical practice where mandatory follow-up angiography is not performed.

## Conclusion

In Korean patients undergoing new-generation DES implantation for coronary artery disease, EES and R-ZES showed similar angiographic outcomes at 9 months and comparable clinical outcomes during 2.8 years of median follow-up. This study was only powered in comparison of angiographic outcome, 9-month in-segment late loss. For the comparison of clinical outcomes, larger study with adequate patient’s number is warranted.

## Competing interests

Financial/nonfinancial disclosures: The authors have reported that no potential conflicts of interest exist with any companies/organizations whose products or services may be discussed in this article.

## Authors’ contributions

Each author has participated in this research as follows. JML - conception and design, data analysis & interpretation, drafting of the manuscript; TJY - conception and design, data analysis & interpretation, critical review of the manuscript, final approval of the manuscript submitted; JJP - critical review of the manuscript; IYO - critical review of the manuscript; CHY - critical review of the manuscript; JWS - data analysis & interpretation, critical review of the manuscript; YSC - critical review of the manuscript; GYC - critical review of the manuscript; IHC - critical review of the manuscript; DJC - critical review of the manuscript, final approval of the manuscript submitted. All authors read and approval the final manuscript.

## Pre-publication history

The pre-publication history for this paper can be accessed here:

http://www.biomedcentral.com/1471-2261/13/65/prepub

## Supplementary Material

Additional file 1: Table S1 Independent Variables Used in the Propensity-Score Model. Table S2. Baseline Clinical and Angiographic Characteristics of the Patients with Follow-up Angiography. Table S3. Detailed Description of Stent Thrombosis. Table S4. Baseline Clinical and Angiographic Characteristics after Propensity Score Matching. Click here for file
