# Supplementary material for: Comparison of 9-month angiographic outcomes of Resolute zotarolimus-eluting and everolimus-eluting stents in a real world setting of coronary intervention in Korea
Source: BMC Cardiovasc Disord. 2013 Sep 8;13:65. doi: 10.1186/1471-2261-13-65 (PMC3846488; doi:10.1186/1471-2261-13-65)
Supplement: Additional file 1: Table S1 — Independent Variables Used in the Propensity-Score Model. Table S2. Baseline Clinical and Angiographic Characteristics of the Patients with Follow-up Angiography. Table S3. Detailed Description of Stent Thrombosis. Table S4. Baseline Clinical and Angiographic Characteristics after Propensity Score Matching. [file 1471-2261-13-65-S1.doc]

**Additional File**

Title : Comparison of 9-month angiographic outcomes of Resolute zotarolimus-eluting and everolimus-eluting stents in a real world setting of coronary intervention in Korea

Joo Myung Lee, MD^a^, Tae-Jin Youn, MD, PhD^b^, Jin Joo Park, MD^b^, Il-Young Oh, MD, PhD^b^, Chang-Hwan Yoon, MD, PhD^b^, Jung-Won Suh, MD, PhD^b^, Young-Seok Cho, MD, PhD^b^, Goo-Yeong Cho, MD, PhD^b^, In-Ho Chae, MD, PhD^b^, Dong-Ju Choi, MD, PhD^b^

- **Table S1.** Independent Variables Used in the Propensity-Score Model.
- **Table S2.** Baseline Clinical and Angiographic Characteristics of the Patients with Follow-up Angiography.
- **Table S3.** Detailed Description of Stent Thrombosis.
- **Table S4.** Baseline Clinical and Angiographic Characteristics after Propensity Score Matching.

**Table S1.** Independent Variables Used in the Propensity-Score Model.

| **Variables** | **Measure** |
| --- | --- |
| Age | Continuous |
| Sex (M or F) | Nominal |
| Hypertension | Nominal |
| Diabetes mellitus | Nominal |
| Current smoking | Nominal |
| Dyslipidemia | Nominal |
| Chronic renal failure | Nominal |
| Acute myocardial infarction (< 72 hours) | Nominal |
| Previous PCI | Nominal |
| Previous CABG | Nominal |
| Previous myocardial infarction | Nominal |
| Previous cerebrovascular accident | Nominal |
| Left main coronary artery procedure | Nominal |
| Left anterior descending coronary artery procedure | Nominal |
| In-stent restenosis | Nominal |
| Chronic total occlusion | Nominal |
| Bifurcation^†^ | Nominal |
| Type B2 or C lesions^‡^ | Nominal |
| Long lesion (lesion length ≥ 36 mm) | Nominal |
| Small vessel (reference diameter ≤ 2.75 mm) | Nominal |
| Severe left ventricular dysfunction  (Left ventricular ejection fraction < 30%) | Nominal |
| Primary PCI to acute ST-segment elevation MI | Nominal |
| Multivessel procedure (2 or more vessel stenting) | Nominal |

^†^ Bifurcation means bifurcated lesion that have been treated solely by drug-eluting stents.

^‡^ Type B2 or C lesions according to ACC/AHA classification.

Abbreviations: CABG, coronary artery bypass graft; MI, myocardial infarction; PCI, percutaneous coronary interventions.

**Table S2**. Baseline Clinical and Angiographic Characteristics of the Patients with Follow-up Angiography.

|  | **EES (N= 264)** | **R-ZES (N= 181)** | **P value** |
| --- | --- | --- | --- |
| **Clinical Characteristics** |  |  |  |
| Age, year | 62.18 ± 11.57 | 63.53 ± 10.99 | 0.217 |
| Male | 200 (75.8%) | 133 (73.5%) | 0.587 |
| Diabetes mellitus | 106 (40.2%) | 65 (35.9%) | 0.366 |
| Hypertension | 186 (70.5%) | 111 (61.3%) | 0.052 |
| Dyslipidemia | 89 (33.7%) | 58 (32.0%) | 0.713 |
| Cerebrovascular disease | 20 (7.6%) | 13 (7.2%) | 0.876 |
| Peripheral artery disease | 2 (0.8%) | 5 (2.8%) | 0.095 |
| Chronic renal failure | 5 (1.9%) | 9 (5.0%) | 0.068 |
| Current smoker | 201 (76.1%) | 130 (71.8%) | 0.306 |
| Previous PCI | 45 (17.0%) | 24 (13.3%) | 0.278 |
| Previous CABG | 2 (0.8%) | 2 (1.1%) | 0.703 |
| Previous MI | 1 (0.4%) | 2 (1.1%) | 0.607 |
| Clinical Indication |  |  |  |
| Stable angina | 128 (48.5%) | 90 (49.7%) | 0.797 |
| Unstable angina | 62 (23.5%) | 41 (22.7%) | 0.838 |
| Acute myocardial infarction | 74 (28.0%) | 50 (27.6%) | 0.925 |
| Emergency PCI for acute STEMI | 39 (14.8%) | 24 (13.3%) | 0.887 |
| Left ventricular ejection fraction | 57.64 ± 9.79 | 56.38 ± 11.67 | 0.274 |
| Severe LV dysfunction (LVEF < 30%) | 2 (0.8%) | 5 (2.8%) | 0.095 |
| Multivessel disease | 165 (62.5%) | 129 (71.3%) | 0.055 |
| **Angiographic chracteristics** |  |  |  |
| Target vessel location |  |  |  |
| Left main artery | 15 (5.7%) | 6 (3.3%) | 0.247 |
| LAD | 145 (54.9%) | 89 (49.2%) | 0.233 |
| LCX | 52 (19.7%) | 42 (23.2%) | 0.373 |
| RCA | 66 (25.0%) | 49 (27.1%) | 0.624 |
| Type B2 or C lesions^†^ | 165 (62.5%) | 114 (63.0%) | 0.917 |
| In-stent restenosis | 23 (8.7%) | 9 (5.0%) | 0.134 |
| Chronic total occlusion | 11 (4.2%) | 9 (5.0%) | 0.687 |
| Bifurcation^‡^ | 9 (3.4%) | 13 (7.2%) | 0.071 |
| Small vessel^§^ | 92 (34.8%) | 77 (42.5%) | 0.100 |
| Long lesion^¶^ | 47 (17.8%) | 37 (20.4%) | 0.485 |

^*^ Data are number (%), unless otherwise indicated. Plus-minus values are means ± SD.

^†^ Type B2 or C lesions according to ACC/AHA classification.

^‡^ Bifurcation means bifurcated lesion that have been treated solely by drug-eluting stents.

^§^ Small vessel denotes lesion with reference diameter ≤ 2.75 mm.

^¶^ Long lesion denotes lesion with length ≥ 36 mm.

Abbreviations: CABG, coronary artery bypass graft; EES, everolimus-eluting stent; LAD, left anterior descending artery; LCX, left circumflex artery; LV, left ventricle; LVEF, left ventricular ejection fraction; MI, myocardial infarction; PCI, percutaneous coronary intervention; RCA, right coronary artery; R-ZES, Resolute zotarolimus-eluting stent; STEMI, myocardial infarction with ST-segment elevation.

**Table S3.** Detailed Description of Stent Thrombosis.

|  | **Days after**  **procedure** | **Clinical event** | **Antiplatelet agent at event** |
| --- | --- | --- | --- |
| **Everolimus-Eluting Stent** | | | |
| Probable | 1 | Sudden cardiac death | Aspirin, clopidogrel |
| Definite | 3 | TLR | Aspirin, clopidogrel |
| Definite | 9 | STEMI, TLR | Aspirin, clopidogrel |
| Definite | 166 | STEMI, TLR | Aspirin, clopidogrel |
| Definite | 1079 | STEMI, TLR | Aspirin |
| **Resolute Zotarolimus-Eluting Stent** | | | |
| Probable | 6 | Sudden cardiac death | Aspirin, clopidogrel |
| Definite | 461 | STEMI, TLR | Aspirin, clopidogrel |
| Definite | 727 | STEMI, TLR | None^†^ |
| Probable | 821 | STEMI, cardiac death | Aspirin |

Abbreviations: STEMI, myocardial infarction with ST-segment elevation; TLR, clinically indicated target lesion revascularization.

^†^ Aspirin, clopidogrel stopped for 10 days due to radical prostatectomy for prostate cancer.

**Table S4.** Baseline Clinical and Angiographic Characteristics after Propensity Score Matching.^*^

|  | **EES (N= 249)** | **R-ZES (N= 249)** | **Standardized**  **Differences, %** |
| --- | --- | --- | --- |
| **Clinical Characteristics** |  |  |  |
| Age, year | 65.03 ± 11.48 | 64.86 ± 11.73 | 1.46 |
| Male | 177 (71.1%) | 179 (71.9%) | 1.77 |
| Diabetes mellitus | 100 (40.2%) | 93 (37.3%) | 5.96 |
| Hypertension | 160 (64.3%) | 161 (64.7%) | 0.84 |
| Dyslipidemia | 82 (32.9%) | 83 (33.3%) | 0.85 |
| Cerebrovascular disease | 19 (7.6%) | 18 (7.2%) | 1.53 |
| Peripheral artery disease | 6 (2.4%) | 8 (3.2%) | 4.85 |
| Chronic renal failure | 11 (4.4%) | 12 (4.8%) | 1.91 |
| Current smoker | 72 (28.9%) | 69 (27.7%) | 2.66 |
| Previous PCI | 37 (14.9%) | 39 (15.7%) | 2.22 |
| Previous CABG | 4 (1.6%) | 5 (2.0%) | 3.01 |
| Previous MI | 19 (7.6%) | 25 (10.0%) | 8.48 |
| Indication |  |  |  |
| Stable angina | 100 (40.2%) | 101 (40.6%) | 0.82 |
| Unstable angina | 51 (20.5%) | 52 (20.9%) | 0.99 |
| Myocardial infarction | 74 (29.7%) | 82 (32.9%) | 6.90 |
| Emergency PCI for acute STEMI | 29 (11.6%) | 31 (12.4%) | 2.46 |
| Left ventricular ejection fraction | 57.20 ± 11.12 | 56.23 ± 11.79 | 8.46 |
| Severe LV dysfunction (LVEF < 30%) | 5 (2.0%) | 6 (2.4%) | 2.73 |
| Multivessel disease | 190 (76.3%) | 185 (74.3%) | 4.64 |
| **Angiographic chracteristics** |  |  |  |
| Target vessel location |  |  |  |
| Left main artery | 11 (4.4%) | 8 (3.2%) | 6.28 |
| LAD | 118 (47.4%) | 118 (47.4%) | 0.00 |
| LCX | 66 (26.5%) | 58 (23.3%) | 7.41 |
| RCA | 64 (25.7%) | 71 (28.5%) | 6.30 |
| Type B2 or C lesions^†^ | 160 (64.3%) | 160 (64.3%) | 0.00 |
| In-stent restenosis | 13 (5.2%) | 15 (6.0%) | 3.48 |
| Chronic total occlusion | 11 (4.4%) | 10 (4.0%) | 1.99 |
| Bifurcation^‡^ | 12 (4.8%) | 6 (2.4%) | 12.91 |
| Small vessel^§^ | 100 (40.2%) | 101 (40.6%) | 0.82 |
| Long lesion^¶^ | 44 (17.7%) | 53 (21.3%) | 9.10 |

^*^ Data are number (%), unless otherwise indicated. Plus-minus values are means ± SD.

^†^ Type B2 or C lesions according to ACC/AHA classification.

^‡^ Bifurcation means bifurcated lesion that have been treated solely by drug-eluting stents.

^§^ Small vessel denotes lesion with reference diameter ≤ 2.75 mm.

^¶^ Long lesion denotes lesion with length ≥ 36 mm.

Abbreviations: CABG, coronary artery bypass graft; EES, everolimus-eluting stent; LAD, left anterior descending artery; LCX, left circumflex artery; LV, left ventricle; LVEF, left ventricular ejection fraction; MI, myocardial infarction; PCI, percutaneous coronary intervention; RCA, right coronary artery; R-ZES, Resolute zotarolimus-eluting stent; STEMI, myocardial infarction with ST-segment elevation.
